# Perspectives of health care professionals and policymakers on antimicrobial stewardship and implementation challenges in low- and middle-income country hospital settings: a qualitative systematic review protocol

**DOI:** 10.11124/JBIES-25-00203

**Published:** 2026-02-05

**Authors:** R. Rajalakshmi, M. Kavya, Swapna Nayak, Shruthi Bhandarkar, Jayaraj Mymbilly Balakrishnan, Sreedharan Nair, Sohil Khan, Karattuthodi Mohammed Salim, Sneh Shalini, R. Uday Kumar, N. V. Vajid, Girish Thunga

**Affiliations:** 1 Department of Pharmacy Practice, Manipal College of Pharmaceutical Sciences, Manipal Academy of Higher Education, Manipal, Karnataka, India; 2 JBI Manipal Centre for Toxicovigilance and Drug Safety, Manipal College of Pharmaceutical Sciences, Manipal Academy of Higher Education, Manipal, Karnataka, India; 3 Department of Emergency Medicine, Kasturba Medical College and Hospital, Manipal Academy of Higher Education, Manipal, Karnataka, India; 4 School of Pharmacy and Pharmacology, Quality Use of Medicines Network, Menzies Health Institute, Griffith University, Gold Coast, QLD, Australia; 5 Division of Development Research, Indian Council of Medical Research, New Delhi, India; 6 Department of Pharmacy Practice, Amrita School of Pharmacy, Amrita Vishwa Vidyapeetham, Kochi, Kerala, India; 7 Department of Research and Development, IQRAA International hospital and Research Centre, Calicut, Kerala, India; 8 Centre for Toxicovigilance and Drug Safety, Manipal College of Pharmaceutical Sciences, Manipal Academy of Higher Education, Manipal, Karnataka, India

**Keywords:** antimicrobial resistance, antimicrobial stewardship, barriers, facilitators, infection prevention and control

## Abstract

**Objective:**

The objective of this review is to synthesize qualitative evidence on the perceptions of health care professionals and policymakers regarding barriers and facilitators to implementing antimicrobial stewardship (AMS) programs in hospital settings, particularly in low- and middle-income countries.

**Introduction:**

AMS programs are widely recognized as critical interventions to combat antimicrobial resistance and optimize the use of antibiotics. While quantitative studies have demonstrated the effectiveness of AMS programs in reducing inappropriate antibiotic use, less is known about the experiences, perceptions, and contextual factors that influence their implementation and sustainability.

**Eligibility criteria:**

This review will include qualitative studies exploring the experiences, perceptions, attitudes, and contextual factors influencing implementation of AMS programs among health care professionals and policymakers. Studies conducted in any health care setting and involving any population relevant to AMS programs will be considered.

**Methods:**

The key information sources to be searched for this review will include PubMed, CINAHL (EBSCOhost), PsycINFO (Ovid), Scopus, Embase, and Web of Science Core Collection. The search will include studies published in any language from 2015 onward. Study selection will be conducted independently by 2 reviewers, with any disagreements resolved through discussion or consultation with a third reviewer. All included studies will be critically appraised for methodological quality, and all will proceed to synthesis, regardless of the results of methodological quality assessment. Data relevant to the review objectives will be extracted in terms of both findings and illustrations, followed by an aggregative data synthesis approach. Confidence in the findings will be assessed using a structured grading process based on credibility and dependability.

**Review registration:**

PROSPERO CRD420251044164

## Introduction

Antimicrobial resistance has emerged as one of the most pressing global health challenges, driven largely by inappropriate and excessive use of antimicrobials in both human and animal health.^1^ The excessive and inappropriate use of antibiotics in fields such as agriculture, health care, and livestock expedited the emergence of superbugs (antibiotic-resistant strains). These drug-resistant strains affect treatment efficacy and can lead to severe infections. In addition, progress in the development of newer antibiotics is inadequate, with the risk of the rate of antimicrobial resistance surpassing the identification of effective therapy.^2^ Antimicrobial resistance is linked to increased morbidity and mortality, prolonged hospital stays, higher health care costs, treatment failures, and the spread of multidrug-resistant infections.^3^ These adverse outcomes underscore the urgent need for antimicrobial stewardship (AMS) interventions, in line with the World Health Organization (WHO) recommendations, to preserve the effectiveness of existing antibiotics and safeguard public health.^4^,^5^

AMS programs are a coordinated set of strategies and interventions designed to promote the appropriate use of antimicrobials to improve patient outcomes, reduce microbial resistance, and decrease unnecessary costs.^6^ AMS programs aim to optimize antimicrobial use by promoting selection of the appropriate agent, dose, duration, and route of administration to achieve the best clinical outcomes while minimizing toxicity and resistance.^7^ Despite growing advocacy for AMS implementation, particularly in hospital settings, there is considerable variability in how these programs are interpreted, executed, and sustained, especially in low- and middle-income countries (LMICs).^8^ Promoting education and public awareness is a key strategy of AMS to combat antimicrobial resistance. Health care professionals play a central role in this effort and must have a thorough understanding of AMS activities and core strategies. In hospital settings, AMS programs primarily involve implementing context-specific guidelines, providing targeted education to health care providers, and restricting the use of certain antibiotics to ensure judicious prescribing.^9^ Given the widespread use of antibiotics and the slow pace of new drug development, it is crucial to preserve existing antibiotics while controlling infection rates through comprehensive stewardship initiatives, aligning with the overarching goals of the WHO.^4^,^9^

Health care practices, perceptions of authority, and decision-making processes vary significantly across different cultural and regional contexts. In many LMICs, hierarchical decision-making structures can influence antimicrobial prescribing. Cultural norms related to patient expectations and clinical autonomy may also affect prescribers’ willingness to adopt stewardship guidelines.^10^ Additionally, in LMICs, the concept of stewardship itself may be variably understood or may lack resonance due to competing clinical priorities and limited awareness of antimicrobial resistance. In resource-constrained hospitals, the absence of reliable microbiological diagnostics, limited formularies, and erratic drug supply further complicate adherence to stewardship principles. These local realities underscore the importance of exploring implementation challenges through the lived experiences of health care providers.^11^ Hospitals are often primary drivers of antimicrobial use, especially broad-spectrum antibiotics, and serve as critical points for stewardship intervention.^12^,^13^ Moreover, the hospital environment is complex and influenced by hierarchical decision-making, resource constraints, infection-control practices, and organizational culture—all factors that shape the effectiveness of AMS programs.^14^ Focusing on LMICs acknowledges the global disparity in AMS program infrastructure and implementation.

While AMS programs are well-established in many high-income countries such as England, Norway, and France, LMICs often lack institutionalized guidelines, trained personnel, and robust surveillance systems.^15^,^16^ Resource constraints and health care workforce capacity are key contextual factors influencing the establishment of AMS programs across health care systems. In the United States, AMS programs are structured and mandated by the Infectious Diseases Society of America and the Centers for Disease Control and Prevention (CDC), and focus on leadership commitment, drug expertise, accountability, continuous monitoring, education, and feedback mechanisms.^17^ This emphasizes the development of a multidisciplinary team consisting of an infectious disease physician and clinical pharmacist along with a microbiologist, nursing staff, epidemiologists, and information specialists.

According to the CDC, AMS programs focus on the core strategies, including prospective audits with feedback, pre-authorization of restricted antimicrobials, educational interventions, dose optimization, and switching intravenous-to-oral conversions.^18^ According to reports, a team dedicated to infectious diseases supplemented by hospital administration support considerably reduced the inappropriate use of antimicrobials by 20% to 40%, highlighting the clinical and economic benefits despite the long-term effect on resistance being harder to quantify.^19^ Moreover, the US model mainly focuses on data-driven interventions where hospitals are connected to the National Healthcare Safety Network, which strengthens the surveillance mechanism.^20^ The US model of the AMS program framework has been adapted to several other countries as a comprehensive framework consisting of regulation, surveillance, accountability, and cooperation. A qualitative study by Turner *et al*.^21^ using the COM-B model in the United Kingdom identified organizational support, AMS leadership, support by strong regulatory frameworks, surveillance systems, and education initiatives as critical facilitators of AMS program implementation.

In Australia, AMS program implementation is mandated across all health care settings and is supported by national guidelines and a robust audit mechanism. Centralized surveillance of bacterial pathogens is coordinated through the Australian Group on Antimicrobial Resistance, which integrates automated surveillance systems and voluntary public reporting. National-level bodies, such as the Joint Expert Technical Advisory Committee on Antibiotic Resistance and the Expert Advisory Group on Antimicrobial Resistance, further reinforce policy coordination and strategic planning.^22^ These initiatives demonstrate Australia’s strong national commitment to AMS programs, focusing on key elements such as formulary restrictions, prospective audit and feedback, prescriber education, and continuous monitoring and surveillance by AMS teams in alignment with regulatory policies and as part of the hospital accreditation system.

In contrast, a qualitative study by Charani *et al*.,^16^ examining contextual determinants of AMS programs across low-, middle-, and high-income countries, highlighted that LMICs such as India struggle with underdeveloped infrastructure, limited health care expenditure, and an inadequate workforce to meet rising health care demands. Access to antimicrobials also remains a critical challenge in LMICs such as India, where restricted availability has fostered black-market trade and unauthorized supply. Moreover, the absence of stringent regulatory policies enables antimicrobials to reach the public through multiple informal channels. These findings were reinforced by a qualitative study on the implementation of hospital AMS programs in LMICs from a multiprofessional perspective within the Global Point Prevalence Survey (Global-PPS) network, which emphasized the importance of adopting behavioral approaches and leveraging existing frameworks to strengthen quality improvement programs.^23^ These limitations make it imperative to understand local perspectives and challenges in implementing stewardship interventions.

While there is a growing body of quantitative research on the efficacy and outcomes of AMS interventions, the success of these programs often depends on human and organizational factors that are best explored through qualitative research. Understanding the perspectives of health care professionals is crucial, as they are responsible for the day-to-day functioning of AMS programs. Qualitative research can illuminate these contextual, cultural, and systemic challenges that either support or hinder effective AMS implementation.^24^ The decision to include only qualitative studies in this review is based on the need to capture in-depth, nuanced insights into professional experiences and perceptions that quantitative methods may not reveal. Qualitative evidence can help explain why certain AMS practices succeed or fail, reveal context-specific strategies, and uncover the relational and behavioral aspects of implementation that are often overlooked in broader analyses.^12^

A preliminary search of PROSPERO, PubMed, the Cochrane Database of Systematic Reviews, and *JBI Evidence Synthesis* located qualitative studies conducted in LMICs that explore health care professionals’ experiences with AMS implementation.^13^ These studies are published across a variety of disciplines, including infectious disease, public health, health systems research, and pharmacy practice. Some have been conducted as stand-alone qualitative inquiries, while others are embedded in larger mixed methods evaluations of AMS initiatives. The availability of such studies supports the feasibility of this review and its potential to generate meaningful, context-sensitive insights.

Existing reviews have predominantly focused on AMS effectiveness or implementation in high-income settings, with limited synthesis of qualitative evidence specific to LMIC hospital contexts.^10^,^13^ Thus, this review aims to synthesize qualitative evidence on the barriers and facilitators of AMS as perceived by health care professionals in hospital settings, with a specific focus on LMICs. These regions face unique constraints, such as limited infrastructure, resource scarcity, variable health literacy among patients, and weaker regulatory enforcement, all of which influence AMS uptake and sustainability.^11^ This review will focus on health care professionals and policymakers involved in antimicrobial use and stewardship in hospital settings. These individuals are critical to the operational success of AMS programs and include physicians, pharmacists, nurses, infectious disease specialists, and microbiologists. Including a range of health care professionals allows for a more comprehensive understanding of the multidisciplinary nature of AMS. This review will fill a critical gap by highlighting how health care professionals and policymakers in these settings interpret and operationalize stewardship efforts, as well as the structural and cultural constraints they navigate.

## Review questions

How do health care professionals and policymakers perceive the implementation of AMS programs in hospital settings LMICs?

Additional questions include:

- What are the perceptions, attitudes, and experiences of health care professionals and policymakers regarding the implementation of AMS programs in hospital settings?
- What are the perceived facilitators and barriers to the effective implementation of AMS programs in hospital settings, as experienced by health care professionals and policymakers?

- What contextual factors, such as organizational culture, policies, and interprofessional dynamics, influence health care professionals’ and policymakers’ engagement with AMS programs across primary, secondary, and tertiary hospital settings?

## Eligibility criteria

### Participants

This review focuses on health care professionals and administrators/policymakers who are directly or indirectly involved in the implementation and delivery of AMS programs within hospital settings. Eligible health care professionals include physicians, pharmacists, nurses, microbiologists, and infection prevention and control specialists, provided they are formally registered to practice within their scope. Hospital administrators and policymakers responsible for policy enforcement and resource allocation related to AMS programs will also be included.^25^ These individuals play a critical role in antimicrobial prescribing, surveillance, infection control, and policy enforcement, making their insights essential to understanding the practical realities of AMS implementation.

### Phenomena of interest

The central phenomena of interest are the perceptions, attitudes, and lived experiences of health care professionals and policymakers regarding the implementation of AMS programs. Guided by a constructivist epistemology perspective, this review recognizes that knowledge, awareness, and perceptions are contextually constructed through the professional and institutional experiences of these stakeholders rather than objectively measured. The studies that explicitly state they are AMS will be included in the review to avoid the subjective bias of interpretation. Specifically, the review seeks to identify and explore the barriers and facilitators to effective engagement with AMS, as well as the broader contextual factors that shape implementation outcomes. *Barriers* are factors that hinder or obstruct the implementation of AMS programs, and *facilitators* are enablers or supportive factors. The constructs for the implementation factors of AMS programs will be identified based on Consolidated Framework of Implementation Research (CFIR).^26^ These include elements such as organizational culture, institutional policies, and interprofessional dynamics, which can significantly influence how AMS practices are adopted and sustained in routine clinical care.

### Context

The review is situated within hospital settings across various levels of care, including primary, secondary, and tertiary hospitals. The geographical focus is on LMICs, classified as per the World Bank and based on gross national income per capita.^27^ Countries categorized as low income, lower-middle income, or upper-middle income at the time of the review will be included. These countries have limited resources and health care facilities, and may have unique systemic, cultural, and infrastructure challenges that directly impact the implementation and effectiveness of AMS programs.^28^ Given that LMICs often face resource constraints, inconsistent access to diagnostics, and variable adherence to prescription guidelines, it is especially important to understand how contextual influences can impact how health professionals engage with AMS programs in these countries.

### Types of studies

This review will consider qualitative studies that explore the perceptions, experiences, attitudes, and contextual understandings of health care professionals and policymakers regarding the implementation of AMS programs in hospital settings. Eligible studies may employ a range of qualitative methodologies, including phenomenology, grounded theory, ethnography, action research, and feminist research.

The review will include interpretive studies that draw on the lived experiences of health care professionals involved in antimicrobial prescribing, infection control, and AMS implementation, as well as critical studies that explore power dynamics, institutional structures, and sociocultural influences affecting AMS engagement and practice.

Studies that use qualitative methods for data collection and analysis, such as interviews, focus groups, and participant observations, will be included, regardless of whether they are stand-alone qualitative studies or the qualitative component of a mixed methods design, provided that the qualitative findings are clearly reported and distinguishable.

## Methods

The proposed systematic review will be conducted in accordance with the JBI methodology for systematic reviews of qualitative evidence,^29^,^30^ and reported in line with the Preferred Reporting Items for Systematic Reviews and Meta-Analyses (PRISMA) guidelines.^31^ This protocol has been registered with PROSPERO (CRD420251044164).

### Search strategy

The search strategy will aim to locate both published and unpublished studies. A 3-step search strategy will be utilized in this review. First, an initial limited search of PubMed and Embase was undertaken to identify articles on the topic. The text words contained in the titles and abstracts of relevant articles, along with the index terms used to describe those articles, were analyzed to develop a comprehensive search strategy for PubMed (Appendix I). The search strategy, including all identified keywords and index terms, will be adapted for each included database/information source. The reference lists of all included sources of evidence will be screened for additional studies, specifically the reference lists of studies included in the systematic review and those of relevant systematic reviews on similar topics.

Tools such as Yale MeSH Analyzer (Yale University, New Haven, USA) may be used to identify relevant keywords and indexing patterns. No search filters, including methodological filters, date restrictions, or language limits, will be applied during the initial phase to ensure maximum sensitivity and comprehensiveness. Studies published in any language will be included, and studies published in languages other than English will be translated using tools such as Microsoft Translator (Microsoft, Washington, USA). Translations will be verified by a language expert to ensure accuracy and reliability.

Studies published since 2015 will be included, as the concept of AMS programs came into existence during that period, making the research most relevant to current clinical and policy contexts. Information sources to be searched will include the following electronic databases: PubMed, CINAHL (EBSCOhost), PsycINFO (Ovid), Scopus, Embase, and Web of Science Core Collection. In addition, sources of unpublished studies and gray literature to be searched will include ProQuest Dissertations and Theses Global, Trove (National Library of Australia), Google Scholar (first 200 results), relevant conference proceedings, and professional organizational websites related to antimicrobial resistance/AMS programs. Finally, authors of included studies or conference abstracts will be contacted up to 2 times when clarification or unpublished data are required. If no response is received, the study will still be included, but any missing data will be noted and accounted for in the analysis, as appropriate.

### Study selection

Following the search, all identified citations will be collated and uploaded into EndNote v.21 (Clarivate Analytics, PA, USA) and duplicates removed. Prior to commencing study selection, all reviewers will undertake a structured training protocol designed to promote consistency and rigor in the screening process. This training will include familiarization with the eligibility criteria and a pilot-screening exercise on a subset of studies. Following the pilot test, titles and abstracts will then be screened by 2 reviewers (KM and SB) independently for assessment against the eligibility criteria for the review.

Potentially relevant studies will be retrieved in full and their citation details imported into the JBI System for the Unified Management, Assessment and Review of Information (JBI SUMARI; JBI, Adelaide, Australia).^32^ The full text of selected citations will be assessed in detail against the eligibility criteria by 2 reviewers (KM and SN) independently. Reasons for exclusion of full-text papers that do not meet the eligibility criteria will be recorded and reported in the systematic review. To assess the reliability of reviewer decisions, inter-reviewer agreement will be measured using Cohen’s kappa statistics, with established interpretation thresholds. For studies with poor agreement (κ < 0.40) between reviewers, the decision to include or exclude the study will be discussed and decided through consensus or consultation with a third reviewer (RR or GT). The results of the search and the study inclusion process will be reported in full in the final systematic review and presented in a PRISMA flow diagram.^31^

### Assessment of methodological quality

Eligible studies will be critically appraised by 2 reviewers (SN and KM) independently for methodological quality using the JBI Critical Appraisal Checklist for Qualitative Research.^33^ This tool includes 10 items assessing key domains such as congruity between research methodology and the research question, data collection methods, representation of participant voices, researcher influence, and ethical considerations. Each item on the checklist will be rated as “yes,” “no,” “unclear,” or “not applicable.”

A pilot test, similar to that conducted for study selection, will be performed to ensure consistency prior to quality appraisal. Two reviewers (RR and KM) will randomly select a subset of included articles and independently apply the critical appraisal tool criteria. Their assessments will be recorded, and the level of agreement will be measured using Cohen’s kappa statistics: κ > 0.70 will be considered acceptable, indicating a high level of agreement between the authors. Any discrepancies will be discussed and resolved through mutual consensus or discussion with the third reviewer (RR or GT) before proceeding with the full appraisal. No automation tools will be used in the methodological quality assessment process. Authors of the papers will be contacted twice to request missing or additional data for clarification, where required. Any disagreements that arise between the reviewers will be resolved through discussion or with a third reviewer (RR or GT).

The results of the critical appraisal will be reported both narratively and in tabular format. To determine the rating for each checklist item, a response will be rated:

- positive (”yes”) if the study provides sufficient and relevant information to confidently support the item
- negative (”no”) if the study clearly lacks or contradicts the criteria for the item
- unclear if the information provided is insufficient or ambiguous.

All studies, regardless of their methodological quality, will undergo data extraction and synthesis, where possible.

### Data extraction

Data will be extracted from studies included in the review by 2 reviewers independently (KM and SB) using a modified JBI data extraction tool (Appendix II) for qualitative research in JBI SUMARI.^32^ If necessary, the tool will be modified to better align with the specific objectives and scope of this review. Modifications may include the addition of fields to capture review-specific contextual variables or emergent themes not originally included in the standard template. Any such modifications will be documented and appended as supplementary material.

Equity, diversity, and inclusion data will be captured using the PROGRESS-plus framework (place of residence, race/ethnicity/culture/language, occupation, gender/sex, religion, education, socioeconomic status, social capital, plus other relevant factors) to assess uniform representation and identify underrepresentation, as well as highlight potential inequities.^34^ In addition, the key implementation factors influencing AMS programs in health care settings will be identified and categorized based on the constructs of CFIR.^26^ The authors will extract the data when the studies explicitly mention cultural and contextual factors and analyze it narratively; where data are not available, it will be highlighted as an evidence gap.

To minimize errors during data extraction, a calibration exercise will be conducted prior to full extraction on a subset of selected studies to ensure consistency and clarity in the use of the tool. Extracted data will be cross-checked by both reviewers. Any discrepancies will be resolved through discussion or, where needed, arbitration by a third reviewer (RR or GT). Findings and their illustrations will be extracted and assigned a level of credibility: unequivocal, credible, or not supported. Findings will be defined as the authors’ analytical interpretations or conclusions derived from the data. Illustrations will refer to the supporting evidence from the original study (eg, participant quotations or excerpts) that substantiate the findings. Findings will be rated as unequivocal when they are accompanied by illustrations that are clearly and contextually aligned with, and directly support, the interpretation. A credible rating will be assigned to findings supported by illustrations within the manuscript but where the connection between the evidence and the interpretation may be less explicit or detailed. Findings will be rated as not supported when they lack any accompanying illustrations or when the available evidence does not adequately support the stated finding.

If required, authors of included papers will be contacted by email correspondence or, where feasible, by telephone to request missing or additional data for clarification up to 3 times. In the case of missing or unclear information, the review team will make reasonable assumptions based on contextual information provided within the paper. All such assumptions will be transparently reported in the review.

### Data synthesis

Qualitative research findings will, where possible, be pooled using JBI SUMARI with the meta-aggregation approach.^29^ This will involve the aggregation or synthesis of findings to generate a set of statements that represent that aggregation through assembling the findings and categorizing these findings based on similarity in meaning. These categories will then be subjected to a synthesis to produce a single comprehensive set of synthesized findings that can be used as a basis for evidence-based practice. Where textual pooling is not possible, the findings will be presented in narrative format. Only unequivocal and credible findings will be included in the synthesis, while unsupported findings will be excluded to maintain the methodological rigor of the review.

### Assessing confidence in the findings

The final synthesized findings will be graded according to the ConQual approach for establishing confidence in the output of qualitative research synthesis and presented in a Summary of Findings.^35^ The Summary of Findings will include the major elements of the review and detail how the ConQual score is developed. Included in the Summary of Findings will be the title, population, phenomena of interest, and context for the review. Each synthesized finding from the review will then be presented, along with the type of research informing it, scores for dependability and credibility, and the resulting overall ConQual score.

## Acknowledgments

Manipal Academy of Higher Education, Manipal College of Pharmaceutical Sciences, and the Department of Pharmacy Practice for their academic and infrastructural support throughout the conduct of this review. The Department of Health Research, Indian Council of Medical Research (ICMR) for guidance in methodological aspects, and to the IQRAA International Hospital and Research Centre for facilitating access to relevant clinical resources and expertise.

## Author contributions

The concept for the review was devised by GT. Conduct of the preliminary search and development of the protocol was done by RR. The initial search was conducted by KM, SN, SB. The protocol was reviewed by JMB, SN, MSKT, SK, UR, VNV, SS. All authors approved the final version of the manuscript.

## Appendix I: Search strategy

**Table:**

PubMed		
Search conducted: October 15, 2025		
Search	Query	Records retrieved
#1	((Stewardship, Antimicrobial) OR (Antibiotic Stewardship)) OR (Stewardship, Antibiotic)	17,278
#2	(((((((((((Drug Resistances, Microbial) OR (Antibiotic Resistance, Microbial)) OR (Antibiotic Resistance)) OR (Resistance, Antibiotic)) OR (Antimicrobial Drug Resistance)) OR (Antimicrobial Drug Resistances)) OR (Antimicrobial Resistance, Drug)) OR (Antimicrobial Resistances, Drug)) OR (Drug Antimicrobial Resistance)) OR (Drug Antimicrobial Resistances)) OR (Resistance, Drug Antimicrobial)) OR (Resistances, Drug Antimicrobial)	293,382
#3	Control, Infection	503,065
#4	((((Attitudes) OR (Opinions)) OR (Opinion)) OR (Sentiment)) OR (Sentiments)	991,877
#5	((((Behaviors) OR (Acceptance Processes)) OR (Acceptance Process)) OR (Process, Acceptance)) OR (Processes, Acceptance)	5,343,666
#6	Epistemology	1,185,725
#7	((((((((Awarenesses) OR (Situational Awareness)) OR (Awarenesses, Situational)) OR (Awareness, Situational)) OR (Situational Awarenesses)) OR (Situation Awareness)) OR (Awarenesses, Situation)) OR (Awareness, Situation)) OR (Situation Awarenesses)	380,533
#8	Knowledge [MeSH Terms]	15,638
#9	(((Perception) OR (Perceptions, Social)) OR (Social Perceptions)) OR (Perception, Social)	829,838
#10	Physician	887,950
#11	(((((((((((Personnel, Health) OR (Healthcare Workers)) OR (Healthcare Worker)) OR (Health Care Providers)) OR (Health Care Provider)) OR (Provider, Health Care)) OR (Healthcare Providers)) OR (Healthcare Provider)) OR (Provider, Healthcare)) OR (Health Care Professionals)) OR (Health Care Professional)) OR (Professional, Health Care)	1,158,683
#12	((Infection Control Practitioner) OR (Practitioner, Infection Control)) OR (Practitioners, Infection Control)	4603
#13	((Medical Staffs) OR (Staff, Medical)) OR (Staffs, Medical)	142,558
#14	((((((((((((((((Hospital Medical Staffs) OR (Staff, Hospital Medical)) OR (Staffs, Hospital Medical)) OR (Medical Staffs, Hospital)) OR (Hospital Medical Staff)) OR (Attending Physicians, Hospital)) OR (Attending Physician, Hospital)) OR (Hospital Attending Physician)) OR (Hospital Attending Physicians)) OR (Registrars, Hospital)) OR (Hospital Registrar)) OR (Hospital Registrars)) OR (Registrar, Hospital)) OR (Physicians, Junior)) OR (Junior Physician)) OR (Junior Physicians)) OR (Physician, Junior)	105,979
#15	((((((Nurse) OR (Nursing Personnel)) OR (Personnel, Nursing)) OR (Registered Nurses)) OR (Nurse, Registered)) OR (Nurses, Registered)) OR (Registered Nurse)	574,001
#16	((((((((((((Pharmacist) OR (Clinical Pharmacists)) OR (Clinical Pharmacist)) OR (Pharmacist, Clinical)) OR (Pharmacists, Clinical)) OR (Community Pharmacists)) OR (Community Pharmacist)) OR (Pharmacist, Community)) OR (Pharmacists, Community)) OR (Retail Pharmacists)) OR (Pharmacist, Retail)) OR (Pharmacists, Retail)) OR (Retail Pharmacist)	58,530
#17	((#1) OR (#2)) OR (#3)	776,260
#18	(((((#4) OR (#5)) OR (#6)) OR (#7)) OR (#8)) OR (#9)	7,124,483
#19	((((((#10) OR (#11)) OR (#12)) OR (#13)) OR (#14)) OR (#15)) OR (#16)	2,081,107
#20	((#17) AND (#18)) AND (#19)	23,836
Limited to 2015-2025		13,987

## Appendix II: Draft data extraction tool

**Table:**

Study details	Author, year, country of origin
Population characteristics	participant demographics, sample size, recruitment method
Contextual information	setting, cultural background, cultural norms, geographic location
Study methodology	design, data collection methods, data analysis approach
EDI factors	guided with PROGRESS-plus framework
Phenomena of interest	as defined by the review objective
Key findings relevant to the review question, along with illustrative quotes or examples	
The assigned level of credibility for each finding, based on the 3-level JBI categorization	unequivocal, credible, or not supported

EDI, equity, diversity, and inclusion.
